# Implementing point-of-care medical information systems into trauma and general surgeon practice in a middle-income country: a qualitative study utilizing the Consolidated Framework for Implementation Research

**DOI:** 10.1186/s43058-023-00397-4

**Published:** 2023-04-06

**Authors:** Stephanie N. Wilson, Helen Noble, Willy Jesús Neumann Ordoñez, Gabriela Zavala Wong, Manuel J. Rodríguez, David Ortega Checa, Maria Warne, Kirsten Senturia, Lacey Nicole LaGrone

**Affiliations:** 1 Applied Worldwide, Boulder, CO USA; 2 Northern Pacific Global Health Fogarty International Program, University of Washington, Seattle, WA USA; 3 Sociedad de Cirujanos Generales del Perú, Lima, Perú; 4 Medical School, Universidad Peruana Cayetano Heredia, Lima, Perú; 5 Department of Surgery, Universidad Peruana Cayetano Heredia, Lima, Perú; 6 Department of Surgery, Hospital Rebagliati, Lima, Perú; 7 University of Colorado Health, Denver, CO USA; 8 University of Washington, Seattle, WA USA; 9 Department of Surgery, Medical Center of the Rockies, University of Colorado Health, Loveland, CO USA

## Abstract

**Background:**

Point-of-care medical information systems (POCMIS) can provide an efficient and effective means of strengthening health systems globally through their encouragement of continued medical education. Using the Consolidated Framework for Implementation Research (CFIR) as a guide, this research provides suggestions for improved implementation of POCMIS in low- and middle-income countries informed by an intervention implemented across public and military hospitals in Lima, Peru.

**Methods:**

Analysis is based on qualitative interviews conducted with 12 Peruvian surgeons across eight public hospitals and one military hospital who received an intervention that provided free access to UpToDate and introduced Google Translate. The post-intervention interviews were transcribed, translated, and analyzed for themes overlapping with CFIR constructs to expose barriers to implementation and suggestions for improved implementation of future interventions.

**Results:**

Barriers included a lack of seniority buy-in and engaged leadership, an overabundance of personal preferences for multiple POCMIS, and a culture of assumption that inhibited open communication regarding access to and use of POCMIS. Suggestions for improved implementation focused on the adaptation of the intervention. Namely, surgeons discussed regionally-specific adaptations as well as adaptations specific to their surgical specialty including visual, rather than written, representation of the information available via POCMIS.

**Conclusions:**

Results indicate necessary adaptations for implementing interventions including POCMIS in LMICs, mimicking much of the implementation science literature on intervention adaptation. In addition to explicit suggestions provided by surgeons, we also suggest actionable steps to adapt to barriers identified in our data. Rapid assessment procedures (RAP) are one established methodological technique useful for assessing organization culture prior to implementation, allowing for necessary cultural adaptations. Dynamic adaption process (DAP) is another useful and established method that breaks implementation into four phases allowing for adaptations based on the initial assessment of the intervention site.

**Supplementary Information:**

The online version contains supplementary material available at 10.1186/s43058-023-00397-4.

Contributions to the literature

- Point-of-care medical information systems (POCMIS) such as UpToDate have been shown to improve patient outcomes. Yet, evidence to guide the implementation of these resources for surgeons in low- and middle-income and non-English speaking countries is limited.
- Through qualitatively evaluating the implementation of POCMIS for surgeons in nine hospitals in Lima, Peru, we provide insight on how the implementation process can be adapted and improved for future endeavors.
- Our findings bring to light important cultural and specialty-specific factors influencing the implementation of POCMIS that have yet to be discussed in the literature.

## Background

Quality improvement of surgical care in low- and middle-income countries (LMICs) has been a World Health Organization priority since establishing the Global Initiative for Emergency and Essential Surgery Care in 2005 [1]. Given the reported link between the use of information systems encouraging evidence-based medicine and improved patient outcomes [2], such information systems are of interest across resource settings. One cost-effective path to global health equity is building strong surgical systems [3, 4], and continued medical education (CME) has the potential to strengthen surgical systems. Information systems such as UpToDate (UTD) support CME, suggesting a pathway to stronger surgical systems [5–7].

With medical literature constantly evolving as new research is published, providers often cannot stay current for more than a few years after completing medical school [8]. Evidence from one systematic review shows that increased years of provider practice correlates with lower performance—measured as clinical knowledge and adherence to standards of practice—suggesting providers are less likely to follow updated clinical guidelines the further they are from their postgraduate medical education. This evidence held true across subspecialties, including surgery [9].

Safely applying primary literature to clinical practice is time-consuming [10]. When time is limited, point-of-care medical information systems (POCMIS) may help by efficiently and effectively encouraging CME [5, 11, 12]. POCMIS refer to technological and informational tools that assist providers with clinical decision-making [13]. Studies investigating POCMIS across medical specialties show high use of and satisfaction with POCMIS in healthcare settings globally [14] along with improvement in self-directed e-learning for CME [15].

Despite these known benefits, financial and language barriers to POCMIS remain for healthcare providers in non-English speaking countries and LMICs [16]. Other barriers discussed in the literature include lack of: time, awareness of accessible resources, and information intake capacity, to name a few [17]. In response to these persisting barriers, this short report provides recommendations for how we can encourage effective implementation of POCMIS for surgeons in LMICs.

## Methods

The educational intervention for this research included a didactic on evidence-based practice (EBP) and use of Google Translate, along with provision of application for free UTD access to surgical providers at nine hospitals in Lima, Peru. Participants were interviewed after receiving the intervention and their responses were analyzed for this short report, which conforms to the consolidated criteria for reporting qualitative research (COREQ [See Additional file 1]).

### Intervention

Surgeons practicing in the USA (L.N.L.), Peruvian General Surgery Society board members (M.R., G.B., D.O.C., J.H.), a medical educator (J.L.R.), and graphic designer (W.L.R.) developed a one-hour presentation reviewing theories of EBP from previously published EBP courses [18–22, 22–24], used interactive clinical practice questions alongside UTD articles, and introduced Google Translate. The study team invited 12 of the largest hospitals from the military, public, and social security systems in Lima to participate. Nine of these hospitals ultimately enrolled [25]. After the presentation, participants applied for a grant to receive free, individual access to UTD for one calendar year via the Better Evidence UTD Donations Program [26]. Upon closure of data collection, the control group also received the intervention.

### Data collection

Following the intervention, we conducted qualitative interviews with 10 attending surgeons and two residents. The interview sample consisted of eight general surgeons, two trauma surgeons, one laparoscopic surgeon, and one combined general surgeon/surgical oncologist. Of the 12 providers, three identified as women and nine as men.

We used the Consolidated Framework for Implementation Research (CFIR) to create a semi-structured interview guide focused on how surgeons find information to answer their clinical questions and about their experiences using UTD (see Additional file 2). Interviews were conducted via phone by a Peruvian researcher with an extensive practical background in qualitative research, lasted an average of 41 min, and were recorded on the interviewer’s laptop for transcription and translation by a bilingual researcher.

### Analysis

Data were uploaded into Dedoose Version 7.0.23 (Sociocultural Research Consultants, Los Angeles, California) for coding and analysis by H.E.N. and M.W. following procedures outlined by Braun and Clarke [27]. Transcripts were open coded, coders were blind to each other’s coding, and differences were resolved by discussion until 100% agreement was reached. A second qualitative methodologist (S.N.W.) further analyzed specific code excerpts from the initial analysis to compile findings for this report. See Additional file 3 for detailed methods.

## Results

Findings from the interviews expose barriers to implementation and provide suggestions for improved implementation of the POCMIS intervention. We present those barriers and suggestions using constructs identified in CFIR (18 [see Appendix for CFIR diagram]).

### Barriers to implementation

Thematically, barriers to implementation emerged as three separate themes in the data: seniority buy-in and engaged leadership, personal preference, and culture of assumption. *Seniority buy-in and engaged leadership* relates to the construct of implementation climate within the hospitals. *Personal preference* relates to CFIR’s other personal attributes construct. *Culture of assumption* is explained through CFIR constructs culture and networks and communication. See Table 1 of Additional File 4 for a summary on how barriers identified correlate with CFIR constructs, themes from the analysis, and particularly illustrative quotes from the interviews.

#### Seniority buy-in and engaged leadership

Assumptions specific to senior surgeons and those with authority came through in the interviews as a motivator for action—or inaction—and in doing so exposed implementation climate barriers. For example, about a previous chief of service at their hospital, one surgeon said,

It was different a few years ago when Doctor [redacted] was the chief of service, he was constantly looking up new studies, new information. He guided us and gave us orientation, he motivated us to keep looking up information. We lost all of that since he retired. (INT-01)

Another surgeon further demonstrated the impact individuals have on organizational culture and medical practice when discussing another senior physician, telling us,

I read, double check, and follow what evidence-based medicine says, but unfortunately I live in an “obedience-based medicine” system…my boss says “you have to do this with the patient,” “but doctor, this drainage is not recommended,” “no, you have to do it,” “but literature has shown meta-analysis where this doesn’t work on certain patients,” “I don’t care, you have to do it, I have more experience with many more patients.” (INT-09)

In the same way, an engaging chief of service can motivate colleagues to continue learning and growing as surgeons, a leader resistant to change can impede growth. As yet another surgeon put it,

Sometimes the senior workers are more reluctant to change…They don’t accept the changes that come with technology. It’s a constant battle. It gets a little tedious because they take it personally. (INT-04)

Notably, the same interviewee who commented on an association between seniority and reluctance to change acknowledged the value of collaboration with more experienced partners in certain clinical scenarios: “If I’m dealing with a difficult case, I usually turn to more experienced people who can give me some advice on how to solve the problem.” (INT-04).

#### Personal preference

When discussing surgeons’ preferences during interviews, we were met with a long list of personal preferences for a variety of search engines, including UTD, PubMed, Google, YouTube, and more. For instance, one surgeon told us, “it’s up to each other’s preferences. Some are still using the regular system (unintelligible), others have PubMed. It depends on what they like” (INT-10). Providers having pre-established preferences for POCMIS made universal adoption of the intervention challenging.

#### Culture of assumption

Cultural barriers and networks and communication barriers came through in the data as one theme: *culture of assumption*. For example, one surgeon told us “I think it’s useful to have this technology handy. If they didn’t have the motivation to use it before, they have it now, *I believe* they have it now.” (INT-08). When followed up with a question about what search engines colleagues use, the surgeon then said, “we have talked about it when [the researcher] came here, she did a presentation, but I couldn’t say,” referring to the presentation from the intervention.

This surgeon’s response indicates a common theme among interviewees’ assumptions about POCMIS culture among their colleagues, i.e., a culture of assumption, rather than a culture of discussion. Take the following interview excerpt:

P: ...They say “ok, I’m going to do it!,” but I don’t ask them later if they did it or not. It’s usually a conversation in the moment and I don’t do a follow up later, like “hey, did you check this?, did you login on UpToDate?” None of that.

I: You didn’t overhear any comments if they did it either?

P: Not really. We haven’t talked about that. (INT-07).

### Suggestions for improved implementation

In addition to demonstrating barriers to implementation, our interviews with surgeons also revealed practical suggestions for improved implementation. We present explicit suggestions in the results section while extrapolating further suggestions from the data in the discussion section. See Table 2 in Additional file 4 for a summary on how suggestions correlate with CFIR constructs, themes from the analysis, and particularly illustrative quotes from the interviews.

#### Suggested adaptations

Adaptability was a prominent area for suggested improvements throughout the data. Suggestions for improved adaptability touch on two areas: regionally-specific adaptations and specialty-specific adaptations.

##### Regionally specific adaptations

Adaptations related to the specific region of practice are vital based on interview responses. For example, one surgeon told us, “Since we’re here in Lima, we do have resources to diagnose patients, but we fall short when it’s about treatment. We don’t have too many resources.” (INT-09). Similarly, another surgeon mentioned how the evidence they find through POCMIS may not be applicable to their region:

Since they are taken from literature produced in other countries, I’ve found out about other treatment options and even though it doesn’t apply to what I do here, now I know about new and different treatments I could use if they were available here, mostly in the surgical field. (INT-07)

One participant also noted how financial resources differ by region, saying “probably in the United States [UTD is] affordable, but here it’s a lot of money” (INT-03).

##### Specialty specific adaptations

Participants also noted their desire for more visual presentation of information via POCMIS, rather than written information with one surgeon, saying “compared to other medical specialties…we go for another type of information: graphic and visual information” (INT-06). Another surgeon told us, “Solutions for surgical problems are not written down and I can tell you that [from] experience” (INT-04). Surgeons also noted the uniqueness of the production of medical knowledge in surgical specialties saying, “experiments cannot be performed on human beings and…a complex surgery performed in an animal cannot be extrapolated to a human being” (INT-09).

## Discussion

The research presented here aims to provide a roadmap for future interventions focused on increasing access to POCMIS for surgeons in LMICs. Because CFIR was developed in a high-income context, applying it to LMICs often requires adaptation [28]. In line with that literature, our results highlight the importance of adaptability for low-resource settings. Other researchers have similarly noted the need to adapt surgery-focused interventions—as well as the frameworks used for implementation—for LMICs, such as Zambia, Brazil, and Benin [29, 30].

Also important in our findings were specialty-specific adaptations, which took surgeons’ desires for visual and graphic information into account. With attention to the growing literature on adaptation in implementation science, we suggest our findings on adaptability of this intervention are used to help build an *adaptome* data platform [31] to better understand how deviations from fidelity may lead to better outcomes based on context. Though many surgeons requested more visuals which are easily accessible with limited time, the user experience of UTD was generally positive with participants reporting plans to use UTD in the future as well as positive feedback regarding its ease of use.

### Suggestions for encouraging seniority buy-in and engaged leadership

In addition to explicit recommendations from surgeons themselves, we suggest necessary steps for ensuring seniority buy-in and engaged leadership during implementation. Such efforts may also positively impact the implementation climate by fostering a *learning climate*. Two major components of a *learning climate*—including leaders’ expressing fallibility and a need for colleagues’ assistance, and team members feeling they are a valuable part of the change process—were not clearly identified in the implementation climate. In line with previous literature on implementation [32], ensuring those in leadership roles and with organizational influence support the intervention is an essential first step in ensuring success. Further, considering theories of technology uptake in healthcare settings may be necessary to address these barriers.

### Suggestions for adapting to cultural barriers

In carefully choosing leaders with both formal and informal influence in an organization to champion an intervention, we recognize the power of individuals as carriers of culture and their influence on implementation climate [33]. In doing so, we also recognize culture as an enabling force in addition to the constraining force it can be [34].

To harness culture as the enabling force it has the potential to be, we suggest future implementations begin with rapid assessment procedures (RAP) to assess the organizational culture [35]. As an ethnographic method, RAP is well-situated to assess *culture*, including organizational culture, by using methods such as interviews with key informants and organizational stakeholders. Other researchers have successfully used RAP for implementing health informatics, such as electronic health records [36] and computerized provider order entry [37], in healthcare settings. While researchers have identified limitations of RAP [36], the benefits of introducing RAP to implementation efforts in healthcare settings far outweigh those limitations [38].

Another option for adapting to cultural barriers is the dynamic adaptation process (DAP)—a four-phased process for intervention adaptation that balances intervention fidelity with adaptability [31]. In adopting this approach, implementors can assess organizational culture in phase one of DAP, helping them determine what adaptations will be necessary to prepare prior to implementation.

### Limitations

Toward the end of the study, COVID-19 spread across the world, requiring our team to finish data collection virtually. Therefore, it is reasonable to assume our sample included less representation of ideas across surgeons involved in the intervention. In other words, participants may have been less likely to participate in the qualitative interviews because the global pandemic may have shifted their priorities. Also reasonable is the assumption that participants were rightfully preoccupied by the emerging global pandemic, inhibiting their ability to fully reflect on the intervention. While the interview data we collected are rich and provide important suggestions for improved implementation, we recognize the limitations in our sample.

## Conclusion

With a worldwide agenda for quality improvement of surgical care in LMICs, interventions will continue to be implemented across settings and cultures [1]. Whether those interventions include POCMIS or not, cultural barriers and the adaptability of the intervention will be important factors. This research provides practical advice for improved implementation of those interventions based on one study conducted in Lima, Peru.

### Supplementary Information

**Additional file 1.** Consolidated criteria for reporting qualitative studies (COREQ): 32-item checklist.

**Additional file 2.** Interview Guide.

**Additional file 3.** Detailed Methods.

**Additional file 4.** Detailed Results.

## Data Availability

The qualitative datasets generated and analyzed during the current study are not publicly available given the need for specific context to grasp their usefulness. However, code reports from the qualitative interviews are available from the corresponding author on reasonable request.

## Appendix

Appendix: Consolidated Framework for Implementation Research Diagram [39]

## Abbreviations

POCMIS: Point-of-care medical information systems
LMICs: Low- and middle-income countries
UTD: UpToDate
CME: Continued medical education
CFIR: Consolidated Framework for Implementation Research
PGSS: Peruvian General Surgery Society
EBP: Evidence-based practice
RAP: Rapid assessment procedures
DAP: Dynamic adaptation process
