# Supplementary material for: Implementing point-of-care medical information systems into trauma and general surgeon practice in a middle-income country: a qualitative study utilizing the Consolidated Framework for Implementation Research
Source: Implement Sci Commun. 2023 Apr 6;4:38. doi: 10.1186/s43058-023-00397-4 (PMC10078056; doi:10.1186/s43058-023-00397-4)
Supplement: Supplementary file 1 — Additional file 1. Consolidated criteria for reporting qualitative studies (COREQ): 32-item checklist. [file 43058_2023_397_MOESM1_ESM.pdf]

**Consolidated criteria for reporting qualitative studies (COREQ): 32-item checklist**

Please indicate in which section each item has been reported in your manuscript. If you do not feel an item applies to your manuscript, please enter N/A.

For further information about the COREQ guidelines, please see Tong *et al.*, 2017: https://doi.org/10.1093/intqhc/mzm042

| No. | Item | Description | Section/file |
|---|---|---|---|
| **Domain 1: Research team and reflexivity** | | | |
| Personal characteristics | | | |
| *1.* | Interviewer/facilitator | Which author/s conducted the interview or focus group? | Methods |
| *2.* | Credentials | What were the researcher's credentials? *E.g. PhD, MD* | not reported |
| *3.* | Occupation | What was their occupation at the time of the study? | Additional file 3 |
| *4.* | Gender | Was the researcher male or female? | Additional file 3 |
| *5.* | Experience and training | What experience or training did the researcher have? | Additional file 3 |
| Relationship with participants | | | |
| *6.* | Relationship established | Was a relationship established prior to study commencement? | Additional file 3 |
| *7.* | Participant knowledge of the interviewer | What did the participants know about the researcher? *E.g. Personal goals, reasons for doing the research* | Additional file 3 |
| *8.* | Interviewer characteristics | What characteristics were reported about the interviewer/facilitator? *E.g. Bias, assumptions, reasons and interests in the research topic* | not reported |
| **Domain 2: Study design** | | | |
| Theoretical framework | | | |
| *9.* | Methodological orientation and theory | What methodological orientation was stated to underpin the study? *E.g. grounded theory, discourse analysis, ethnography, phenomenology, content analysis* | Additional file 3 |
| Participant selection | | | |
| *10.* | Sampling | How were participants selected? *E.g. purposive, convenience, consecutive, snowball* | Additional file 3 |
| *11.* | Method of approach | How were participants approached? *E.g. face-to-face, telephone, mail, email* | Methods |
| *12.* | Sample size | How many participants were in the study? | Methods |
| *13.* | Non-participation | How many people refused to participate or dropped out? What were the reasons for this? | not reported |
| Setting | | | |
| *14.* | Setting of data collection | Where was the data collected? *E.g. home, clinic, workplace* | Additional file 3 |
| *15.* | Presence of non-participants | Was anyone else present besides the participants and researchers? | Additional file 3 |

| | | | |
|---|---|---|---|
| *16.* | Description of sample | What are the important characteristics of the sample? *E.g. demographic data, date* | Methods |
| Data collection | | | |
| *17.* | Interview guide | Were questions, prompts, guides provided by the authors? Was it pilot tested? | Additional file 2 |
| *18.* | Repeat interviews | Were repeat interviews carried out? If yes, how many? | N/A |
| *19.* | Audio/visual recording | Did the research use audio or visual recording to collect the data? | Additional file 3 |
| *20.* | Field notes | Were field notes made during and/or after the interview or focus group? | Additional file 3 |
| *21.* | Duration | What was the duration of the interviews or focus group? | Methods |
| *22.* | Data saturation | Was data saturation discussed? | Additional file 3 |
| *23.* | Transcripts returned | Were transcripts returned to participants for comment and/or correction? | not reported |
| **Domain 3: analysis and findings** | | | |
| Data analysis | | | |
| *24.* | Number of data coders | How many data coders coded the data? | Additional file 3 |
| *25.* | Description of the coding tree | Did authors provide a description of the coding tree? | not reported |
| *26.* | Derivation of themes | Were themes identified in advance or derived from the data? | Additional file 3 |
| *27.* | Software | What software, if applicable, was used to manage the data? | Methods |
| *28.* | Participant checking | Did participants provide feedback on the findings? | not reported |
| Reporting | | | |
| *29.* | Quotations presented | Were participant quotations presented to illustrate the themes / findings? Was each quotation identified? *E.g. Participant number* | Results & Addition |
| *30.* | Data and findings consistent | Was there consistency between the data presented and the findings? | Results & Addition |
| *31.* | Clarity of major themes | Were major themes clearly presented in the findings? | Results & Addition |
| *32.* | Clarity of minor themes | Is there a description of diverse cases or discussion of minor themes? | N/A |

**When submitting your manuscript via the online submission form, please upload the completed checklist as a Figure/supplementary file.**

**If you would like this checklist to be included alongside your article, we ask that you upload the completed checklist to an online repository and include the guideline type, name of the repository, DOI and license in the *Data availability* section of your manuscript.**

Developed from: Allison Tong, Peter Sainsbury, Jonathan Craig, Consolidated criteria for reporting qualitative research (COREQ): a 32-item checklist for interviews and focus groups, International Journal for Quality in Health Care, Volume 19, Issue 6, December 2007, Pages 349–357, https://doi.org/10.1093/intqhc/mzm042
