# Supplementary material for: Implementing point-of-care medical information systems into trauma and general surgeon practice in a middle-income country: a qualitative study utilizing the Consolidated Framework for Implementation Research
Source: Implement Sci Commun. 2023 Apr 6;4:38. doi: 10.1186/s43058-023-00397-4 (PMC10078056; doi:10.1186/s43058-023-00397-4)
Supplement: Supplementary file 2 — Additional file 2. Interview Guide. [file 43058_2023_397_MOESM2_ESM.docx]

**INTERVIEW GUIDE**

[ ] is a note for the interviewer, not to say aloud.

** denotes must have questions

*Anything in italics is a probe, meaning it’s not critical to ask.*

**[Aim: To improve understanding of evidence-based practice and use of electronic medical source uptake and implementation facilitators and barriers for surgical teams in Lima, Peru.]**

*[Introduction:]*

Hello, my name is Carla Martínez and I work with the Peruvian General Surgery Society Research Collaborative. My research colleagues, Dr. Lacey LaGrone, Dr. Manuel Rodriguez, and Dr. Ortega Checa, visited your hospital last fall to talk about evidence-based surgery.

I’m talking with you today because you’re a Peruvian surgeon. There are no right or wrong answers to the questions I’ll ask; I just want to hear your opinions. If you don’t want to answer a question just say so. You don’t have to discuss anything you don’t want to. If you feel the need to stop, just let me know.

This interview could take about 30 minutes. If you’d like to continue longer, we can. Will that be OK for you today?

Do you mind if I record the interview? I can’t always write everything down, so when we write the reports on all interviews, we use recordings and notes to understand the full range of ideas we’ve heard. We’ll delete the recordings but keep the transcripts for up to 5 years. Our write-up will include quotes from different participants but not your name or hospital. We protect your confidentiality and privacy as much as we can.

Let’s start with informed consent. Can I send you a text of our consent form? I can also read it for you.

[*Ideas for probes throughout*

-Tell me more…

-What do you mean by that…

-Can you give me an example…]

**We’ll start with a few basic questions:**

[Demographics:]

1. **What is your surgical specialty?
2. **What year were you born?
3. **What is your gender?
4. **How many days a week, on average, do you work at a private hospital?
5. **~~Do you consider yourself academically active with teaching and research? What are your activities?~~ Thinking of the following academic activities… Do you do any of them? (convert into a checklist; could be way too long and vague if they answer openly). Teach? Where? Participate in a surgical society etc? Mentor students? (show document of table with demographic information)

[Rapport:]

**I’d like to hear a little more about your current activities at XX hospital.**

1. When did you finish your specialty training?
2. What is (are) your role(s) at that hospital?
   1. *Surgeon? Attending? Faculty? Resident?*
   2. *Administrative head? Other type of leader?*

**OK. Thinking about your daily work:**

1. **How often do you find yourself caring for patients (in the clinic, hospital ward, or in the operating room) and realize you need advice?
   1. Where do you go for that advice?
   2. Give me a recent example.
      1. [Here we want to hear about participants’ experiences using information sources, without specifically asking about internet resources]
2. **Compared to other resources, how do you think an online resource might be useful?

[Evidence-based search engine questions:]

**Now I’d like to shift to talking about what we call “evidence-based search engines.” When I use that term, I mean websites that summarize many peer-reviewed journal articles into an easy-to-understand guidebook for doctors. Doctors can use these summaries as a way to answer their clinical questions when they’re short on time and need to make decisions quickly.**

1. Had you used evidence-based search engines prior to this study?
   1. [if yes:]
      1. Which one did you use?
      2. What worked for you when you used it?
         1. *Did using the evidence-based search engine change your clinical practice? Change your patient outcomes?*
      3. What didn’t work well?
         1. *After using an evidence-based search engine, can you tell me if you had any difficulty?*
      4. Did you use any others?
         1. [circle back through ii, ii.1, iii, iii.1]
2. Do you think other surgeons in your hospital see a need for these evidence-based search engines? Why or why not?
   1. What are some things they have said?
   2. What are the search engines called that they used?
   3. Do other surgeons feel a need for these search engines?
   4. Do they like using them?
3. **What are some things that would keep a surgeon like you, or your colleagues, from using evidence-based search engines?
   1. *How do you think the hospital culture plays a role?*
   2. *What about language?*
   3. *Do you think level of training impacts it?*
   4. *What about time?*
   5. *Are people are motivated to use these search engines?*
   6. *Do hospitals have the funds to pay for these search engines?*
4. **Now I want you to picture your daily workflow. By workflow I mean your common events, locations, and day-to-day practices that help your work as a surgeon flow smoothly. How well does using evidence-based search engines [or use the specific search engine they use] fit into your workflow?
   1. [Make sure they explain their individual workflow and institutional workflow. If they don’t include one or the other, ask:]
      1. *What about your individual workflow, specifically?*
      2. *What about your institutional workflow, specifically?*
5. What kinds of changes would motivate surgeons to use evidence-based search engines?
   1. [This is for **personal** motivation.]
   2. *What about in your personal practice?*
   3. *What about for your colleagues?*
6. What kinds of changes would make it easier for surgeons to use evidence-based search engines?
   1. [This may be answered with #14, but it is specifically asking about **structural** issues that prevent use of evidence-based search engines.]
   2. *What about for the hospital system you work in?*
   3. *What about in your personal practice?*
   4. *What about for your colleagues?*
7. If we wanted to identify somebody at your hospital to get evidence-based search engines more widely used, who would we speak with?
   1. Who at your hospital?
   2. What is their role?
   3. Why this person?

[UpToDate specific questions:]

**Last year in October or November, we came to your hospital to distribute a survey and talk about evidence-based medicine. I would like to ask you a few questions regarding your participation in this research.**

1. Did you take the pre-survey last spring?
2. What about the post-survey sent to you at the beginning of April?
3. Did you participate in our Evidence-Based Medicine presentation on XXXX, 2019?
4. Did you apply for the Better Evidence grant?
5. Did you receive the grant?
6. **Have you started using UpToDate?
   1. [if yes:]
      1. **How is it going with UpToDate?
         1. *Has it changed your personal practice? How?*
         2. *How about for your colleagues?*
      2. Will you continue using UpToDate? Why or why not?
   2. [if no:]
      1. **Why not?
      2. What do you think would have to change for you to start using it?
7. **If you were in charge and had the budget to do anything you wanted to make sure surgeons had sufficient, high quality information resources, what would you do? What changes would you make?
   1. [This should be very general.]

**That’s everything I have to ask you today.**

1. **Is there anything else you want to tell us about? Is there anything else we should have asked?

**Thank you for being a part of this project and talking with me today! We really appreciate your participation.**
