# Supplementary material for: Implementing point-of-care medical information systems into trauma and general surgeon practice in a middle-income country: a qualitative study utilizing the Consolidated Framework for Implementation Research
Source: Implement Sci Commun. 2023 Apr 6;4:38. doi: 10.1186/s43058-023-00397-4 (PMC10078056; doi:10.1186/s43058-023-00397-4)
Supplement: Supplementary file 3 — Additional file 3. Detailed Methods. [file 43058_2023_397_MOESM3_ESM.docx]

**DETAILED METHODS**

The educational intervention related to this research was developed in partnership with the Peruvian General Surgery Society (PGSS). The intervention included a didactic on evidence-based practice (EBP) and use of Google Translate, along with provision of application for free UTD access to surgical providers at nine hospitals in Lima, Peru. We recruited participants for the intervention at PGSS meetings and through a surgeon champion at each hospital. Participants provided verbal consent and no compensation was given for their time. They were interviewed after receiving the intervention and their responses were collected within a qualitative framework for detailed analysis. This report conforms to the consolidated criteria for reporting qualitative research (COREQ), for which a checklist is provided in additional file 1.

**Study Site and Context**

Situated in the Andean region of South America, Peru is an upper-middle income country (22). Wireless internet was not reliably available at government hospitals during the course of this study. Typically, people used cellular data with a few desktop computers with wired internet available. Disease pathologies in Peru are typical for an upper-middle income country (e.g., leading causes of death include chronic diseases [23]), and many hospitals do not have the necessary resources to meet international goals for surgical systems (24). Members of the PGSS and U.S. partners who have collaborated on prior multi-institution observational studies comprised the study team. The University of Washington and the Universidad Peruana Cayetano Heredia institutional review boards approved this study.

The study team invited 12 of the largest hospitals from the military, public and social security systems in Lima to participate. In the end, nine of those hospitals were enrolled (25). In the intervention group, 5 hospitals initially received the intervention after which 1 hospital withdrew due to administrative barriers. In the control group, 1 hospital did not meet inclusion criteria due to inability to identify a hospital champion and 1 hospital withdrew due to administrative barriers. In Peru, the COVID19 pandemic began in month 6 of the 8-month study. Non-essential workers quarantined at home and didactic sessions and elective surgeries were paused, likely decreasing the response rate to the follow-up of this study as well as participation in qualitative interviews.

**Intervention**

Surgeons practicing in the U.S. (L.N.L.), Peruvian General Surgery Society board members (M.R., G.B., D.O.C., J.H.), a medical educator (J.L.R.), and graphic designer (W.L.R.) developed a one-hour presentation that reviewed theories of EBP from previously published EBP courses (26,27,28,29,31,31,32,33), used interactive clinical practice questions alongside UTD articles, and explained Google Translate. Of the 9 hospital champions, 1 chose to present the slideshow themselves while 8 of 9 chose to have a member of our research team present (G.Z.W., W.N.) (34).

After the presentation, participants applied for a grant to receive free, individual access to UTD for one calendar year via the Better Evidence UTD Donations Program (35). Following the grant application process, we emailed and texted each participant regarding their application and provided links to recommended UTD articles relevant to the survey questions. After closure of data collection, the control group also received the intervention.

**Data Collection**

We conducted qualitative interviews with 12 providers who received the intervention, including 10 attending surgeons and 2 residents. The interview sample consisted of 8 general surgeons, 2 trauma surgeons, 1 laparoscopic surgeon and 1 combined general surgeon/surgical oncologist. Of the 12 providers, 3 identified as women and 9 as men.

We used CFIR to create a semi-structured interview guide focused on how surgeons find information to answer their clinical questions and about their experiences using UTD. Questions were intended to ascertain the context, facilitators, and barriers to using POCMIS (Appendix B). To recruit interview participants, we used email, WhatsApp, and phone calls. All participants who received the intervention were invited to participate.

Interviews were conducted via phone by a Peruvian researcher with an extensive practical background in qualitative research (C.M.). C.M. ​​was a consultant for qualitative research projects at the time of the interview and had prior experience in research interviews. She did not establish a relationship with participants prior to or following the interviews, and participants did not have knowledge of her specific role or goals with the research. Only the interviewer and participant were present for phone interviews. Interviews lasted an average of 41 minutes and were recorded on the interviewer’s laptop for transcription and translation by a bilingual researcher.

**Analysis**

Data were uploaded into Dedoose Version 7.0.23 (Sociocultural Research Consultants, Los Angeles, California) for coding and analysis by H.E.N. and M.W. following procedures outlined by Braun and Clarke (39). A codebook was developed iteratively by deriving initial codes from study goals, instruments and proposals, then augmenting the list through reading two transcripts, followed by testing them on three additional transcripts by both coders, and finally editing the codebook until an exhaustive but manageable code list was reached. Transcripts were open coded, coders were blind to each other's coding, and differences were resolved by discussion until 100% agreement was reached. During synthesis, coded excerpts were systematically summarized into themes and subthemes with associated quotes.

A second qualitative methodologist (S.N.W.) further analyzed specific code excerpts from the initial analysis and synthesis to compile findings for this paper including: views about colleagues, setting, types (search engines), type preference, quality (search engines), reliability/quality/satisfaction with UTD, evidence-based medicine (EBM) advocate, administrative disconnect, and specialty differences. Using CFIR constructs as a guide, S.N.W. thematically analyzed the initial code excerpts with a focus on barriers to implementation and suggestions for improved implementation (36,37,38).
