# Supplementary material for: Implementing point-of-care medical information systems into trauma and general surgeon practice in a middle-income country: a qualitative study utilizing the Consolidated Framework for Implementation Research
Source: Implement Sci Commun. 2023 Apr 6;4:38. doi: 10.1186/s43058-023-00397-4 (PMC10078056; doi:10.1186/s43058-023-00397-4)
Supplement: Supplementary file 4 — Additional file 4. Detailed Results. [file 43058_2023_397_MOESM4_ESM.docx]

**DETAILED RESULTS**

Findings from the interviews expose barriers to implementation and provide suggestions for improved implementation of the POCMIS intervention. We present those barriers and suggestions using constructs identified in CFIR. Barriers include: implementation climate, other personal attributes, culture, and networks and communication. Suggestions include improved adaptability based on region and medical speciality.

**Barriers to Implementation**

Thematically, barriers to implementation emerged as three separate themes in the data. First, we observed what we call *seniority buy-in and engaging leadership,* which relates to the construct of implementation climate and, specifically, the learning climate within the hospitals. The second theme, *personal preference* relates to the other personal attributes construct within CFIRs characteristics of individuals domain. Lastly is the theme we call *culture of assumption*, explained through CFIR constructs culture and networks and communication. See Table 1 for a summary of how barriers identified correlate with CFIR constructs, themes from the analysis, and particularly illustrative quotes from the interviews.

| **Table 1: Barriers to Implementation by CFIR Construct** | | |
| --- | --- | --- |
| CFIR Constructs | Themes | Quotes |
| Implementation Climate (learning climate); Engaging | Seniority Buy-in and Engaging Leadership | 1. “Everything starts with whoever is in charge. If the chief of service doesn’t do their part and encourage people to participate in academic activities, this isn’t going to (unintelligible).” (INT-01) 2. “This problem goes beyond doctors, we’re talking about the entire hospital, everybody should use it (unintelligible) and I think this has to be under the supervision of the Teaching and Education Department. It’s a different person in charge every year, and I think they should be promoting this, it goes exclusively through them. They have the power to reinforce this with academic purposes. They should motivate people, these goals should not be just words, they should be based on something tangible, on trustworthy information available for us. The director doesn’t care much for this, neither does the deputy director. If each department does it individually, there won’t be a standard response.” (INT-02) 3. “As chief, I always thought it was my duty to get the best out of each of them, there might be something that stands out and people can’t see it. That was my job as chief, I had to be with them, stimulate them, motivate them” (INT-03) 4. “It depends on the head of the service. As they say, a good chief has to set the tone. If I were the head of the surgery department, I would be there asking things like ‘what’s your opinion on this?, what’s that based on?.’ That would be a motivation they don’t have now due to this chaotic and crazy world we live in. It’s a way to motivate them. They would realize they should be using these search engines, they need these search engines. That would depend on them.” (INT-04) |
| Other Personal Attributes | Personal Preferences | 1. “The next step requires us to get a search engine of our preference, the first thing we should do, the most convenient one.” (INT-02) 2. “Because each person has different goals, a different approach, different daily routine.” (INT-05) 3. “Each person has their own sources of information. I’m not sure if my colleagues, if the people who work with me use it or not necessarily.” (INT-06) |
| Culture; Networks and Communication | Culture of Assumption | 1. “They don’t use the search engine. Some of them say ‘I don’t know how to use a phone,’ so *I’m assuming they don’t know about the search engine and they don’t use it*. (INT-07) 2. “**I:** Have surgeons or your colleagues or other residents made any specific comments or maybe they have told you about a different search engine other than UpToDate?   **P:** No, not really. We haven’t even talked about UpToDate.  **I:** Ok. So, you haven’t talked about search engines.  **P:** No, no.  **I:** Do you think your colleagues feel comfortable when using search engines?  **P:** They surely do.” (INT-01)   1. “I don’t know if there are funds with the current situation, but it could be suggested at management level, that’s why I couldn’t say more about it. I don’t know, maybe at some point, UpToDate could be available for free for some institutions (unintelligible). I’m not sure, it’s just an idea.” (INT-08) |

*Seniority Buy-in and Engaging Leadership*

The data clearly demonstrated both the importance of seniority buy-in and engaging leadership for successful implementation and the barriers that a lack of these factors introduced. Assumptions specific to senior surgeons and those with authority came through in the interviews as a motivator for action—or inaction—and in doing so exposed implementation climate barriers. For example, about a previous chief of service at their hospital, one surgeon said,

It was different a few years ago when Doctor [redacted] was the chief of service, he was constantly looking up new studies, new information. He guided us and gave us orientation, he motivated us to keep looking up information. We lost all of that since he retired. (INT-01)

Another surgeon further demonstrated the impact individuals have on organizational culture and medical practice when discussing another senior physician when they told us,

I read, double check, and follow what evidence-based medicine says, but unfortunately I live in a “obedience-based medicine” system…my boss says “you have to do this with the patient”, “but doctor, this drainage is not recommended”, “no, you have to do it”, “but literature has shown meta-analysis where this doesn’t work on certain patients”, “I don’t care, you have to do it, I have more experience with many more patients”. That’s how they still think…So in order to avoid getting into arguments with a few colleagues, each surgeon is treating their patients according to their approach. (INT-09)

In the same way an engaging chief of service can motivate colleagues to continue learning and growing as surgeons, a leader resistant to change can impede that growth. As yet another surgeon put it,

Sometimes the senior workers are more reluctant to change…They don’t accept the changes that come with technology. It’s a constant battle. It gets a little tedious because they take it personally. (INT-04)

Notably, the same interviewee who commented on an association between seniority and reluctance to change acknowledged the value of collaboration with more experienced partners in certain clinical scenarios:

If I’m dealing with a difficult case, I usually turn to more experienced people who can give me some advice on how to solve the problem. I ask for the patient’s consent and explain it’s a complex case and I need extra help from another surgeon, I need their opinion, specifically. (INT-04)

*Personal Preferences*

Other individual factors, such as personal preference for certain POCMIS or search engines, also came through in the data as prominent barriers. When discussing surgeons’ preferences during interviews, we were met with a long list of personal preferences for a variety of search engines, including UTD, PubMed, Google, Google Scholar, YouTube, and more. For instance, one surgeon told us “it’s up to each other’s preferences. Some are still using the regular system (unintelligible), others have PubMed. It depends on what they like” (INT-10). With most providers having their own preferences and workflows for POCMIS already established, universal adoption of the intervention was challenging.

*Culture of Assumption*

Cultural barriers and networks and communication barriers came through in the data as one theme: *culture of assumption*. For example, one surgeon told us “I think it’s useful to have this technology handy. If they didn’t have the motivation to use it before, they have it now, *I believe* they have it now.” (INT-08). When followed up with a question about what search engines colleagues use, the surgeon then said, “we have talked about it when [the researcher] came here, she did a presentation, but I couldn’t say,” referring to the presentation from the intervention.

This surgeon’s response indicates a common theme among interviewees’ assumptions about POCMIS culture among their colleagues. In other words, we observed a culture of assumption, rather than a culture of discussion. For instance, take the following interview excerpt:

**P:** ...They say “ok, I’m going to do it!,” but I don’t ask them later if they did it or not. It’s usually a conversation in the moment and I don’t do a follow up later, like “hey, did you check this?, did you login on UpToDate?” None of that.

**I:** You didn’t overhear any comments if they did it either?

**P:** Not really. We haven’t talked about that. (INT-07)

A similar lack of discussion was present when conversing about funding opportunities with participants with the same surgeon saying,

At the clinic, I can talk directly to someone in administration because I feel it’s easier, but I know at a hospital it gets more complicated. I guess the head of service can send a request to the chief and then the chief can talk to administration. I honestly don’t know very well how things work when someone has to ask for funding.” (INT-07)

**Suggestions for Improved Implementation**

In addition to demonstrating barriers to implementation, our interviews with surgeons also revealed practical suggestions for improved implementation. We present explicit suggestions from participants here in the results section while extrapolating further suggestions from the data in the discussion section. Table 2 presents the CFIR construct of adaptability as it relates to themes from the analysis, and particularly illustrative quotes from the interviews.

| **Table 2: Suggestions for Improved Implementation by CFIR Construct** | | |
| --- | --- | --- |
| CFIR Constructs | Themes | Quotes |
| Adaptability | Regionally Specific Adaptations; Specialty Specific Adaptations | 1. “I’m talking about more local content, for example, when I search (unintelligible) I always find something interesting. Another example, if it’s about stomach cancer, I’m also interested on what other Latin American countries are doing, different type of experiences” (INT-02) 2. “Most of the time, I enjoy the images, charts, graphics, pictures. Real images or graphics, good pictures and charts truly enhance the article. I find them very interesting and beneficial for visual learning” (INT-05) 3. “We don’t use articles, we watch videos, for example. We go and watch a video, and we see how other surgeons around the world faced that problem. That’s how we draw conclusions.” (INT-06) |

*Suggested Adaptations*

Adaptability, or the degree to which an intervention can be adjusted according to context, was a prominent area for suggested improvements throughout the data. Suggestions for improved adaptability touch on two areas: regionally specific adaptations and specialty specific adaptations.

1. *Regionally Specific Adaptations*

Adaptations related to the specific region of practice are vital based on interview responses. For example, one surgeon told us, “since we’re here in Lima, we do have resources to diagnose patients, but we fall short when it’s about treatment. We don’t have too many resources.” (INT-09). Similarly, another surgeon mentioned how the evidence they find through POCMIS may not be applicable to their region:

Since they are taken from literature produced in other countries, I’ve found out about other treatment options and even though it doesn’t apply to what I do here, now I know about new and different treatments I could use if they were available here, mostly in the surgical field. (INT-07)

One participant also noted how financial resources differ by region, saying “probably in the United States [UTD is] affordable, but here it’s a lot of money” (INT-03).

1. *Specialty Specific Adaptations*

Participants also explicitly noted their desire for more visual presentation of information via POCMIS, rather than written information with one surgeon saying “compared to other medical specialties, [such as] a medical specialty [that] is more prone to look for this kind of articles, we go for another type of information: graphic and visual information.” (INT-06). Another surgeon told us,

I’m talking about surgery because the clinical area pays more attention to what’s in the literature. Solutions for surgical problems are not written down and I can tell you that [from] experience. (INT-04)

Surgeons also noted the uniqueness of the production of medical knowledge in surgical specialties in that “experiments cannot be performed on human beings and…a complex surgery performed in an animal cannot be extrapolated to a human being” (INT-09).

Providing information in various formats may also help with time constraints that are ever present in medical practice. As one surgeon put it, “no one would say they don’t want the search engine, they know everything. The problem here is the lack of time, that’s why they don’t use it.” (INT-04).
